# pyDarwin machine learning algorithms application and comparison in nonlinear mixed-effect model selection and optimization

**DOI:** 10.1007/s10928-024-09932-9

**Published:** 2024-06-28

**Authors:** Xinnong Li, Mark Sale, Keith Nieforth, James Craig, Fenggong Wang, David Solit, Kairui Feng, Meng Hu, Robert Bies, Liang Zhao

**Affiliations:** 1 Department of Pharmaceutical Sciences, School of Pharmacy and Pharmaceutical Sciences State University of New York at Buffalo, Buffalo, NY USA; 2 Software Product Development, Certara, Radnor, PA USA; 3 Division of Quantitative Methods and Modeling, Office of Research and Standards, Office of Generic Drugs, Center for Drug Evaluation and Research, U.S. Food and Drug Administration, Silver Spring, Montgomery, MD USA; 4 Memorial Sloan Kettering, New York, NY USA

**Keywords:** Pharmacokinetics, Modeling, Machine learning, Genetic algorithm, Bayesian optimization, Random forest

## Abstract

Forward addition/backward elimination (FABE) has been the standard for population pharmacokinetic model selection (PPK) since NONMEM® was introduced. We investigated five machine learning (ML) algorithms (Genetic algorithm [GA], Gaussian process [GP], random forest [RF], gradient boosted random tree [GBRT], and particle swarm optimization [PSO]) as alternatives to FABE. These algorithms were applied to PPK model selection with a focus on comparing the efficiency and robustness of each of them. All machine learning algorithms included the combination of ML algorithms with a local downhill search. The local downhill search consisted of systematically changing one or two “features” at a time (a one-bit or a two-bit local search), alternating with the ML methods. An exhaustive search (all possible combinations of model features, N = 1,572,864 models) was the gold standard for robustness, and the number of models examined leading prior to identification of the final model was the metric for efficiency.

All algorithms identified the optimal model when combined with the two-bit local downhill search. GA, RF, GBRT, and GP identified the optimal model with only a one-bit local search. PSO required the two-bit local downhill search. In our analysis, GP was the most efficient algorithm as measured by the number of models examined prior to finding the optimal (495 models), and PSO exhibited the least efficiency, requiring 1710 unique models before finding the best solution. Additionally, GP was also the algorithm that needed the longest elapsed time of 2975.6 min, in comparison with GA, which only required 321.8 min.

**Supplementary Information:**

The online version contains supplementary material available at 10.1007/s10928-024-09932-9.

## Introduction

The current standard method for PPK model selection is FABE. This approach has been little change for nearly 50 years, despite major advances in essentially all other numerical methods in pharmacometrics. The model selection process would be included in the definition of “optimization”. Specifically, FABE would be described as a “local search” or a “greedy search”. In many ways FABE is comparable to gradient based methods of parameter optimization. Among the fundamental weaknesses of FABE are:

- Typically the search starts at “trivial” model, which is likely very different from the true optimal model, resulting in a risk of local minima.
- A local/greedy search is at risk for missing important interaction between model features, as typically only one feature at a time is examined.

In this work we examine the properties of a number of global search algorithms and compare to a “gold standard” of an exhaustive search.

The process of selecting a population pharmacokinetic or pharmacodynamic model to describe a dataset includes at least two related processes. The first is hypothesis generation. Hypotheses regarding candidate models that may best describe the data are generated. These hypotheses may include:

- Number of compartments (e.g., 1, 2, 3)
- Elimination mechanism (e.g., linear or nonlinear)
- Absorption models (e.g., first order, zero order, absorption lag time)
- Covariate relationships. (e.g., absent, linear, power model)

In the ML algorithm paradigm, this set of hypotheses comprises the “search space”. The search space is an N-dimensional discrete space, where each dimension consists of a set of mutually exclusive options. Each candidate model consists of a set of exactly one option chosen from each dimension.

The search space is analogous to the parameter value search space for a single model. The dimensions of the parameter search space are usually real valued and might include:

- Clearance
- Volume of distribution
- Absorption rate constant
- Residual Error variance

**Fig. 1:**
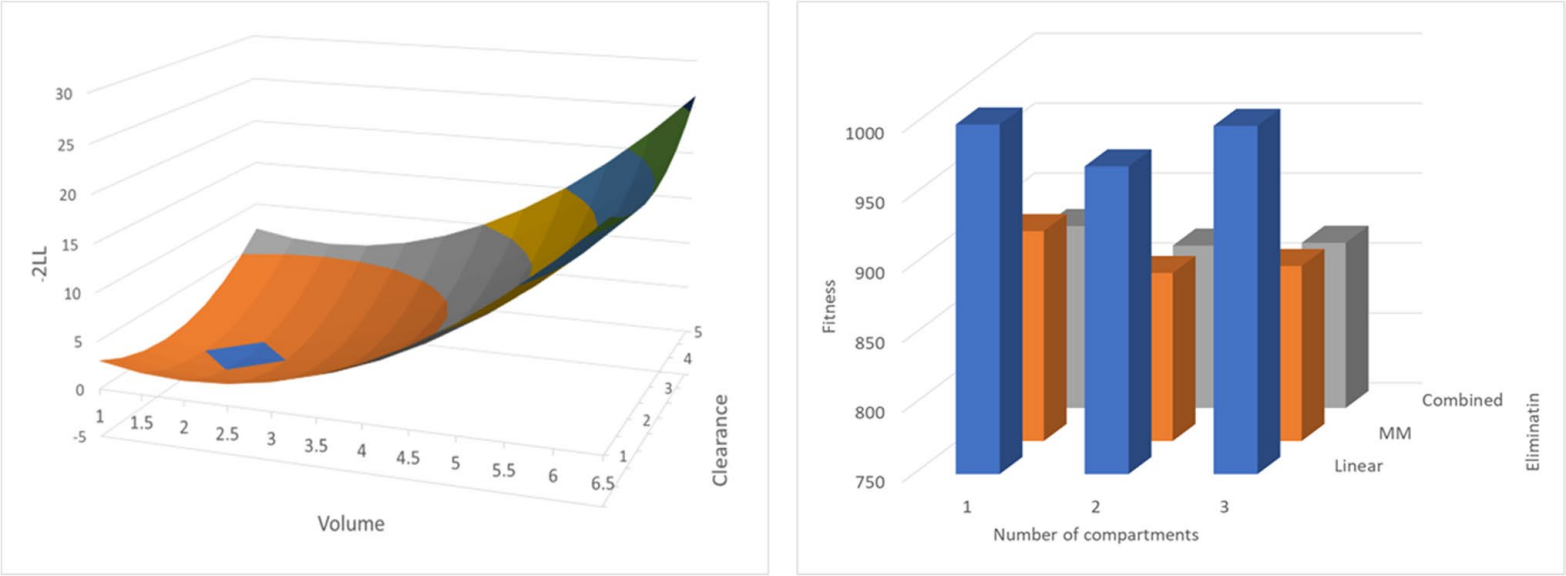
Search space for parameters (left) and model structure (right). *MM is Michael-Menten

The objective in the parameter search space is to find the set of exactly one parameter value in each dimension that optimizes some measure of “model goodness”, often -2 log likelihood (-2LL). A diagram of a (continuous) parameter search space with two dimensions (Volume and Clearance) is given on the left in Fig. 1. The objective in the model structure search space is to find the set of exactly one value in each dimension that optimizes some measure of “model goodness”. A model structure search space with only two dimensions is given on the right in Fig. 1. The (discrete) dimensions of the model structure search space are the elimination mechanism (Linear, Michaelis–Menten, or Combined linear and Michaelis–Menten) and compartment structure (one, two, or three compartments).

The second process in model selection is the selection and testing of the hypotheses generated and comparing models resulting from those hypotheses. That is, what hypothesis to test next and how to decide if that candidate model is a “better” model. In traditional model selection/building, these two processes are undertaken iteratively. Typically, a relatively small set of hypotheses are initially generated based on the understanding of the biology and examination of diagnostic graphics. These are then tested by constructing some of those models, estimating the parameters and examining the results. Criteria for testing the hypotheses typically include both objective (e.g., -2LL) and subjective, including the generation of various diagnostic graphics and consideration of biological plausibility. The generated graphics serve two purposes. First, is the model “good”? For example, does the line, generally, go through the dots? More importantly, graphics serve to generate new hypotheses, which are then tested in subsequent candidate models. The first step in the model selection process—the generation of hypotheses based on the understanding of the biology and graphics belongs to, at the time of this writing, the domain of human intelligence. However, the efficiency and robustness of the selection and testing of hypotheses have recently been expanded to include a range of machine learning methods [1–4] There are several reasons to believe that machine learning methods may be superior to FABE. Interactions between different components of the model leading to local minima in the search space are the most important [5]. In addition, ML model selection can be more objective as decisions regarding biological plausibility, model “goodness” or a “reasonable graphic” defined prior to examination of any results can be subjective. Finally, machine learning based model selection is likely more time efficient, at least with regard to human time, if not computer resource. Note that, as the ML algorithm will not use these subjective evaluations, it remains the responsibility of the human to consider them. The subjective criteria can, and should, be applied before and/or after the objective model search is complete.

A number of numerical algorithms have been proposed for the model selection/hypothesis testing. We will limit our results and discussions to the efficiency and robustness of a subset of these algorithms. Specifically, we will not address the process of hypothesis generation and its potential impact on the final model selection nor the evaluation of graphics. This question has been previously addressed. [6] For the purpose of this analysis, efficiency is defined as the number of evaluations (models run) needed to arrive at the final model and robustness is defined as whether the algorithm can select the optimal model as the final model. The number of evaluations needed to arrive at the final model is used rather than convergence, as there are no generally agreed upon criteria for convergence in these methods. It is assumed that whatever criteria used to define convergence (e.g., 5 generations without change in the model goodness), the number of models run after the optimal model is identified would be consistent across algorithms. For example, if the best model is found at generation 6, the convergence will not be confirmed until generation 11. Therefore, the number of models to find the optimal model is an adequate surrogate for number of models to convergence.

The optimal model in this case was identified by exhaustive search, where every possible combination of the set of hypotheses is run, ensuring that the optimal solution is identified.

## Theoretical

The basic concept of explicitly defining the search space for machine learning model selection, or, for defining it implicitly for traditional model selection has been described previously by Bies [1]. As described above, a multi-dimensional discrete search space is defined, where each dimension represents a set of mutually exclusive model features. In general, these values are strictly categorical (i.e., not ordered categorical). While the options in each dimension will be coded as integers (and therefore ordered categorical), in the algorithms, they are, in all cases, treated as strictly categorical. In the case of GA, the values are recoded as a bit string, reducing them to strictly categorical (values of 0 or 1). Other algorithms have the option to treat the values as strictly categorical and this option is used in all cases.

The options in each dimension are then concatenated into a string of integers. Different algorithms may require different representations of this integer string, such as conversion to a bit string (for GA) or to a “minimal binary” (a binary code without redundancy) string for the one- and two-bit downhill search.

### Algorithms

The algorithms examined include:

- GA – Genetic algorithm attempts to reproduce the mathematics of evolution and survival of the fittest. The algorithm begins by defining a random population (randomly selected bit strings), typically 50–100 models. These bitstrings are translated into NONMEM control files. The resulting NONMEM output is used to calculate the fitness. Models are then randomly selected from the population as candidate “parents”, 2 pairs of 2 models are selected. Each pair undergoes “tournament selection” wherein the model with the better fitness of the pair “wins” and is selected as a parent for the subsequent generation. The two bitstrings of the two parents then undergo crossover and mutation to generate new options. Mutations serve to generate features that have not yet appeared in the population. The Python package DEAP (https://deap.readthedocs.io/en/master/) is used to implement the GA method.
- GP – Gaussian process is implemented with the scikit-learn python package (https://scikit-learn.org/stable/modules/gaussian_process.html). Briefly GP represents the fitness as a generalization of a Gaussian probability distribution. The distribution begins with an uninformative prior. The initial sample is again essentially random, as no information is available to inform it. With the results of that first iteration, the probability distribution is updated. Samples are taken from that updated distribution selected to inform the parameters of the distribution. The models from those samples are then run, and the distribution is updated again. An excellent review of GP can be found here (https://gaussianprocess.org/gpml/chapters/RW.pdf).
- RF and GBRT – Random forest and gradient boosted random tree are implemented using the scikit-learn package (https://scikit-learn.org/stable/modules/ensemble.html). Random forest implements a set of decision trees, classifying the search space based on the fitness values. The multiple trees from random samples (samples, in this case, being the set of models and corresponding fitness) are used to prevent overfitting. Gradient boosted random tree uses a different approach to prevent overfitting. In RF, each tree is independent and created from a random sample of the data. In GBRT, the tree is built additively, with each tree taking the previous, and “boosting” the gradient with respect to fitness by adding some selected low performance models. The addition of low performance models reduces the chances of overfitting as correct classification of poor models is also required.
- PSO – Particle swarm optimization is another attempt to reproduce a natural process, in this case of a flock of birds or a school of fish. The “particles” represent candidate models. As for other algorithms, an initial random population is first created. The movement in the search space is then defined as:

Equation 1. Equation for updating velocity in PSO

1 $${V}_{t+1}={w\centerdot V}_{t}+ {c}_{1}\centerdot {r}_{1}\centerdot (pbest-{x}_{t}) + {c}_{2}\centerdot {r}_{2}\centerdot (gbest-{x}_{t})$$

where V_t+1_ is the velocity at time t + 1, V_t_ is the velocity at time t, ${r}_{1}$ and ${r}_{2}$ are random numbers between 0 and 1, and w is inertia weight (a particle continuing in the same direction), cognitive constant (${c}_{1}$) and social constant (${c}_{2}$) are PSO algorithm parameters, $pbes{t}_{i}$ is the best position that particle *I* has explored, x_t_ is the current particle position, and *gbest* is the best position explored by the entire (global) particle swam. PSO is implemented with the package pySwarms (https://pyswarms.readthedocs.io/en/latest/).

### pyDarwin package

The python package pyDarwin was used for the model selection. Details of the implementation can be found on a public repository on Git Hub (https://github.com/certara/pyDarwin). Details of generation of syntactically correct NONMEM control files from the template and tokens file have been described previously by Bies [1] as well as the pyDarwin documentation (https://certara.github.io/pyDarwin/html/index.html). Briefly, a control file template is created by the user. The control file template is a text file, similar to a NONMEM control file, but with “tokens” that may be replaced to specify features from the search space. Next, a tokens file is created. Both the template and tokens file are identical for all algorithms examined in this analysis. The tokens file is a JSON (Java script object notation) formatted file where each group of sets of key-text pairs represents a dimension of the search space. The sets of key-text pairs correspond to the options in that dimension. The selected element of the key-text pair is substituted into the control file template, creating a syntactically correct NONMEM control file. The control file is then executed with the NMFE{version}.bat command (where {version} is the version number of NONMEM). After execution, the user-defined metric of “model goodness” is calculated. The “model goodness” metric is, for simplicity, called “fitness” for all algorithms, the term used in GA. Other algorithms conventionally use different terms, but we will use “fitness” throughout. Initially, a “population” (again a term from GA) is created with perhaps 50–100 models. When execution of all of these models is complete, the fitness is calculated and the resulting values are used to select the next generation of models. The term “generation” is used throughout to avoid confusion with the term “iteration” used in NONMEM.

### Local downhill search

pyDarwin includes an option for alternating between the ML algorithm and the local downhill search. Including the local downhill search in ML algorithms is from the recognition that while ML algorithms are able to robustly find the area containing global minima, they may struggle in locating the optimal solution within that region. Conversely, local downhill search is expected to find the local minima efficiently. Therefore, a combination of global search and local search strategies will enhance both robustness and efficiency in finding the true optimal solution [10].

For the downhill search, first a “one bit” search is performed. One or more models from the current ML results are used to start the local downhill search. If multiple models are used as the starting point for the one-bit local downhill search, a minimal difference between the models (Hamming distance [7] or niche radius) is defined, so that multiple downhill searches are not started from nearly identical models which would likely lead to the same (potentially local) minimum. Rather, the multiple models are in different “niches”. Each bit in the binary representation of the models is “flipped”, 0 to 1 or 1 to 0 and the resulting models are executed. If any model in the generated set of models is better (has a lower fitness), the process is repeated with the best model. This iteration is continued until no further improvement is seen.

If requested by the user, the one-bit search is followed by a two-bit search. For the two-bit search, the single best model from the one-bit search is used as the base model. All possible combinations of two-bit changes are generated and the resulting models are executed. For illustration, the one and two bit changes for a [0,1;0,0] genome are given in Table 1. Note that the 1 bit changes are on the diagonal and the 2 bit on the off diagonal elements. The first bit change is in columns, and the 2^nd^ in rows. That is, the 2 bit change for the 1^st^ and 2^nd^ bit is given in row 2, column 1 ([1,0;0,0]).

**Table 1:** Table of 1 and 2 bit search genomes for a [0,1;0,0] genome

Change	1^st^ bit	2^nd^ bit	3^rd^ bit	4^th^ bit
1^st^ bit	1,1;0,0			
2^nd^ bit	1,0;0,0	0,0;0,0		
3^rd^ bit	1,1;1,0	0,0;1,0	0,1;1,0	
4^th^ bit	1,1;0,1	0,0;0,1	0,1;1,1	0,1;0,1

**Fig. 2:**
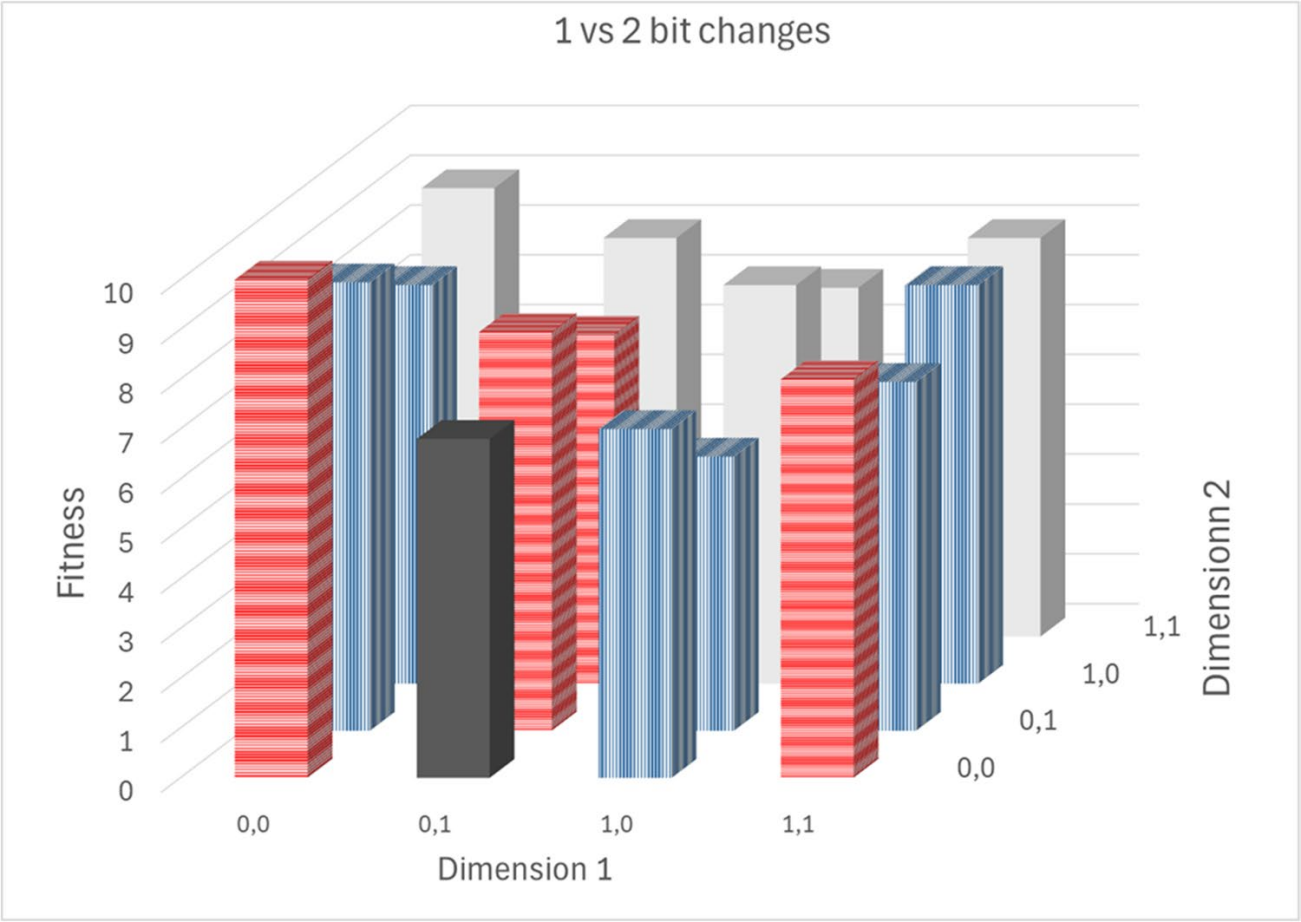
T1 vs 2 bit changes

An illustration of the two-bit search is given in Fig. 2. In this figure, assume that the dark gray genome (0,1 for dimension 1 and 0,0 for dimension 2, i.e., [0,1;0,0]) is the current “best” model after the ML step. The one bit changes from this are shown with horizontal hatching ([0,0;0,0],[0,1;0,1], and [0,1;1,0]. (N.B, [1,0;0,0] is a two bit change despite being adjacent in the figure, with both the 1^s^^t^ and 2^nd^ bit of dimension 1 changed). All of these are worse (higher fitness) than the current best model [0,1;0,0]. Essentially the current “best” [0,1;0,0] is surrounded by a 1 bit wide ridge of worse models. This is a local minimum. Two bit changes (off diagonals Table 1) are shown with vertical hatching. Of these, [1,0;0,1] is has a lower fitness. This model would not be evaluated in a one bit search. Experience suggests this is common in model search spaces. This sort of interaction between model features was first described by Wade et. al [5].

The 2 bit search can be computationally expensive, as the number of models for each iteration is:

Equation 2. Number of models in each two-bit search step

2 $$Number\;of\;models= \frac{n\centerdot (n-1)}{2}$$

where n is the number of bits. In principle the search could be extended to 3 bit changes, but the number of models to be evaluated increases rapidly. These local downhill search steps can be alternated with the ML algorithm at an interval determined by the user. Typically, the downhill period is set as 5, which means after 5 generations of ML search, the local downhill search will begin to run.

## Methods

Five machine learning algorithms were used to select a model for dimethylaminoethylamino-17-demethoxygeldanamycin (DMAG). The clinical study [8] and a population PK model for DMAG has previously been reported [9]. The objective of this analysis was to test whether the algorithms can identify the optimal model, based on the fitness function. For this analysis, the “gold standard” was an exhaustive search, constructing and running all possible models in the search space.

### Data

From 66 subjects, 951 observed concentrations were available. Multiple daily doses were administered, with sample collection for up to 102 h. Ages ranged from 28 to 82 years, weight ranged from 48 to 137 kg with 39% (26 of 66) male and 61% (40 of 66) female. Renal function was normal with the highest serum creatinine of 1.8 mg/dL.

### Search space

The search space, template file and token sets were identical for all algorithms. The search space consisted of 20 dimensions that included the structural model (number of compartments), between subject variability on central volume, peripheral volume, clearance and intercompartment clearance, covariate relationships for central volume, peripheral volume, clearance and intercompartment clearance, between occasion variability on central volume, clearance and intercompartment clearance, and residual error model. A table of the dimensions and options is given in Table 2. In pyDarwin, the search space is coded as token sets, which is given in Supplementary Material 2.

**Table 2:** Search space dimensions

Dimension Name	Options
“ADVAN”	ADVAN1 ADVAN3 ADVAN11
“BSVQ2”	No relationship Log normal between subject variability(BSV) on Intercompartment Clearance 1
“BSVQ3”	No relationship Log normal BSV on Intercompartment Clearance 2
“BSVV2”	No relationship Log normal BSV on Peripheral Volume 1
“BSVV3”	No relationship Log normal BSV on Peripheral Volume 2
“Q ~ WT”	No relationship Intercompartment Clearance 1 a power function of Centered Weight
“Q2 ~ WT”	No relationship Intercompartment Clearance 2 a power function of Centered Weight
“V ~ WT”	No relationship Central Volume a power function of Centered Weight
“V2 ~ WT”	No relationship Peripheral Volume 1 a power function of Centered Weight
“V3 ~ WT”	No relationship Peripheral Volume 2 a power function of Centered Weight
“V ~ AGE”	No relationship Central Volume a power function of Centered AGE
“V ~ SEX	No relationship Central Volume an exponential relationship of SEX (0|1)
“CL ~ SEX”	No relationship Clearance an exponential relationship of SEX (0|1)
“CL ~ WT”	No relationship Clearance a power function of Centered Weight
“CL ~ SCR”	No relationship Clearance a power function of Serum Creatinine
“CL ~ AGE”	No relationship Clearance a power Centered AGE
“IOVCL”	No between occasion variability (BOV) on Clearance Log normal BOV on Clearance
“IOVQ2”	No BOV on Peripheral Clearance 1 Log normal BOV on Peripheral Clearance 1
“IOVV”	No BOV on Volume Log normal BOV on Volume
“RESERR	Combined additive and proportional residual error Proportional residual error only

### Fitness function

The fitness function was identical for all algorithms. The penalties listed in Table 3 were added to the -2LL output from NONMEM. No additional R/Python code penalties were used.

**Table 3:** Penalties

Parameter name	Description	Value
theta	Penalty for each estimated THETA	10
omega	Penalty for each estimated OMEGA element	10
sigma	Penalty for each estimated SIGMA element	10
convergence	Penalty for failing to converge	100
covariance	Penalty for failing the covariance step	100
correlation	Penalty for failing correlation test	100
condition_number	Penalty for condition number > 1000	100

RF, GBRT and GP all use default search options in Table 4. Specific search options used for GA are given in Table 5 and for PSO in Table 6.

**Table 4:** Options for all algorithms

Parameter name	Description	Value
population_size	Number of candidate models in each generation	80
num_parallel	Number of models to run in parallel	32
num_niches	Number of niches to be used	2
niche_radius	Minimum number of bits different between niches (niche radius)	2
downhill_period	Number of ML generations to be run between downhill searches	5
num_generations	Number of generations to be run	20

**Table 5:** GA search parameters

Parameter name	Description	Value
crossover_rate	probability of any pair of parents undergoing cross over	0.95
elitist_num	(number of the best model carried over unchanged to the next generation	4
mutation_rate	probability of any new candidate model undergoing mutation	0.95

**Table 6:** PSO search parameters

Parameter name	Description	Value
cognitive	Parameter for a particle moving toward the best position in its own history	0.9
social	Parameter for a particle moving toward the best position in the populations history	0.8
inertia	Parameter for a particle continuing to move in the same direction	0.5
neighbor_num	Number of nearest neighbors to be used for calculating the population best	2
p_norm	Value of 2, specified using Euclidean distances for calculations	2

The files needed for ML model selection are template file (Supplementary Material 1), tokens.json file (Supplementary Material 2), and options file (Supplementary Material 3). The hardware environment for ML search is based on Intel Xeon Gold 6148 processor at 2.40 GHz with 40 cores in total. Parameter estimation was performed with NONMEM® (ICONplc) version 7.5 with Intel Fortran compiler version 16.0. The first order conditional method with interaction was used for estimation The final parameter estimates for the global optimal model are given in Table 7.

**Table 7:** Parameter values of the optimal model

Parameter	Population estimates (SE%)	Between-subject variability (SE%)	Between-occasion variability (SE%)
CL (L/h)	8.54 (7)	50.3% (11)	30.3% (13)
Q2 (L/h)	79.3 (6)	-	28.6% (13)
Q3 (L/h)	9.74 (13)	75.8% (10)	-
V1 (L)	29.1 (8)	42.9% (25)	31.1% (35)
V2 (L)	71.9 (8)	58.7% (11)	-
V3 (L)	136 (12)	-	-
Power relationship between weight and volume	1.31 (26)	-	-
Proportional error	13.2 (7)	-	-
Additive error (mg/L)	14.7 (39)	-	-

## Results

The search space consists of 1,572,864 candidate m odels. The true optimal model was identified by the exhaustive search. This model included:

- 3 compartments
- Power function of centered WT for volume
- BSV on central volume, clearance, peripheral volume 1, inter-compartment clearance 2
- Between occasion variability on central volume, clearance and intercompartment clearance 1
- Combined additive and proportional residual error model

The parameter values for the final model is reported in the table below:

The control file for this model is provided in Supplementary Material 4. The output file for this model is provided in Supplementary Material 5.

### Pharmacokinetic prediction

**Fig. 3:**
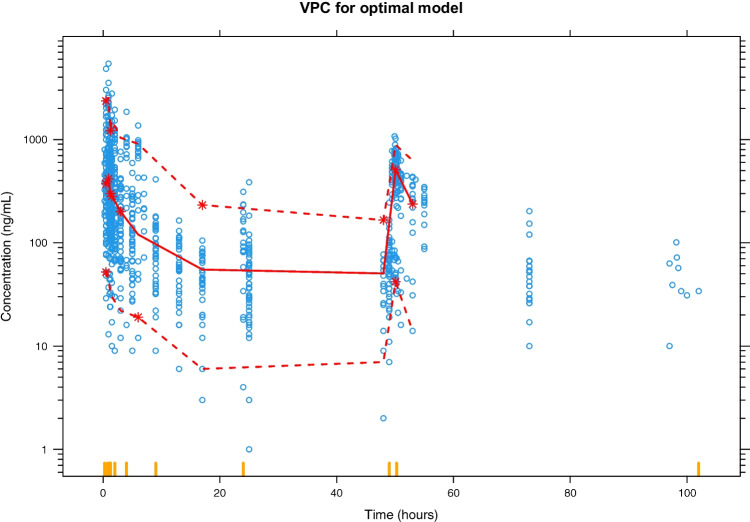
Visual predictive check for the optimal model searched by exhaustive search. Open blue circles are observed DMAG plasma concentrations. The solid red line is the median of observation and the upper and lower dashed red lines are 95^th^ and 5^th^ percentiles of the observed concentrations. The shaded area from top to bottom are 95^th^, 50^th^, and 5^th^ percentiles of the simulation

A visual predictive check for the final model is given in Fig. 3. Despite the optimal model based on the defined search space underpredicted the peak concentrations, it exhibits a generally good prediction of the DMAG pharmacokinetic profile.

### Algorithm comparisons

Table 8 shows the summary results of different ML algorithms. All algorithms were able to identify the optimal model with the two-bit local downhill. With only a one-bit local downhill search, PSO failed to find the optimal model. When evaluating the efficiency of various algorithms, GP was found to be the most efficient, finding the optimal model after examining 495 models. However, GP exhibited decreased efficiency when measured by the metrics of total compute time, requiring 2975.6 min. Notably, according to the time needed per number of unique models generated prior to identifying the true optimal solutions, GP took the longest mean time to generate a single model, compared with GA, RF, PSO, and GBRT, which showed comparable performances. The reason for the longer time for GP to complete is that the computational load for updating and sampling from the posterior distribution increases dramatically as the sample size (total number of models run) increases. GA, and PSO model selection times are independent of the number of models run, as they only use the current population of models for model selection. RF and GBRT do use all available models for models selection, and the bootstrap/bagging for RF has some slow down, but little compared to GP.

**Table 8:** Summary of results by algorithm

Algorithms	Best fitness (OFV + penalties) with one- and two-bit local downhill search	Best objective function value (OFV) with one- and two-bit local downhill search	Time to best model with one- and two-bit local downhill search	Number of unique models to the best model with one- and two-bit local downhill search	Elapsed time with one- and two-bit local downhill search	Best fitness with only one-bit local downhill search	Time (min)/unique model number before reaching the optimal
Exhaustive Search*	8201.271	8041.271	34.2 min	97706	60384.1 min	n/a	0.0035
Genetic Algorithm (GA)	8201.271	8041.271	169.3 min	1307	321.8 min	8201.271	0.130
Random Forest (RF)	8201.271	8041.271	126.7 min	880	461.6 min	8201.271	0.144
Gaussian Process (GP) rerunning	8201.271	8041.271	114.6 min	495	2975.6 min	8201.271	0.232
Particle Swarm Optimization (PSO)	8201.271	8041.271	255.5 min	1710	404.2 min	8220.745	0.150
Gradient Boosted Random Tree (GBRT)	8201.271	8041.271	223.8 min	1328	410.2 min	8201.271	0.169

*Exhaustive search did not include a one- or two-bit downhill search

Note that the exhaustive search spent a short time in getting the optimal model. This is simply a function of the sequence of the token sets in the tokens file. Under other circumstances, this number might be lower than any other machine learning algorithms, while it cannot be regarded as the most efficient.

**Fig. 4:**
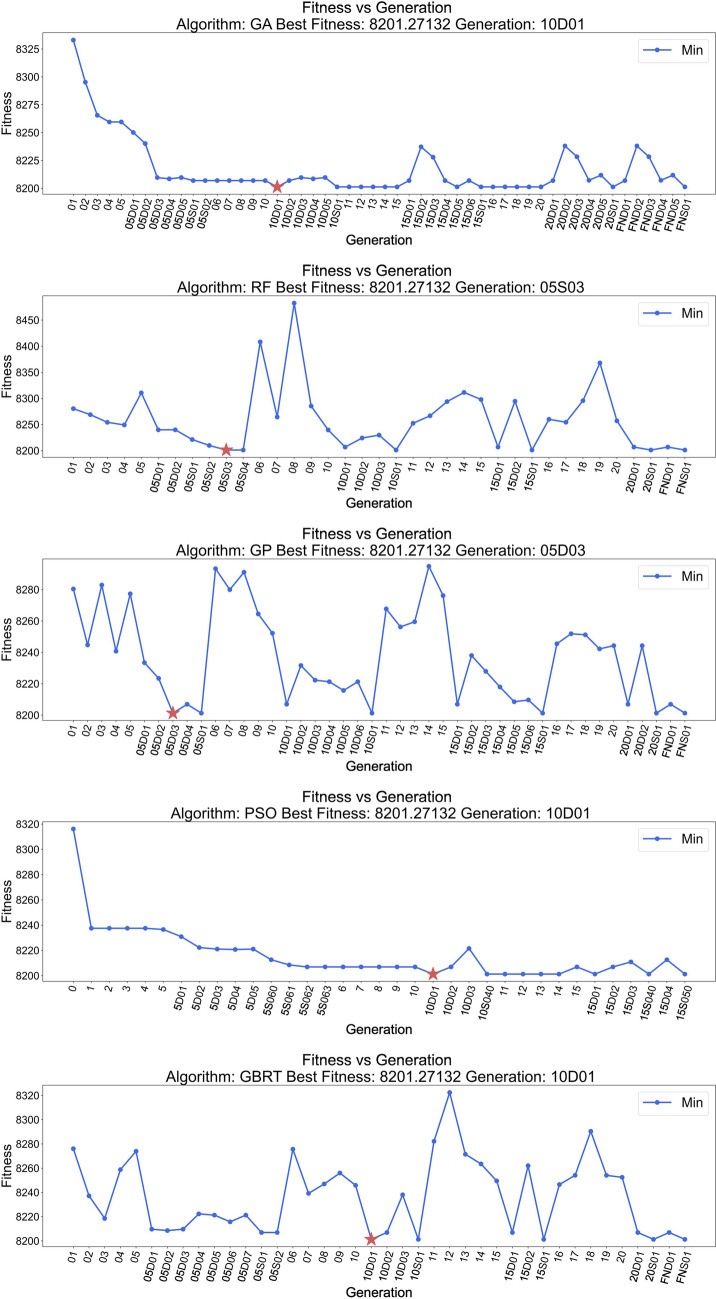
Minimum fitness vs generation by algorithm

Figure 4 shows the minimum fitness by generation under different ML algorithms. GA, PSO, and GBRT found the optimal solution (star symbol) all at the first downhill step after 10 generations, RF was able to find the optimal solution at the third 2-bit search generation after running 5 generations of ML search. GP exhibited the best efficiency by identifying the global minima at the third downhill step after 5 generations of ML search.

The final model describe by pyDarwin can be compared to the final model found in the original analysis [8]. Note that this comparison is limited in that the criteria for model selection were different. The work by Aregbe et al. used a penalty of 3.84 points per parameter for a likelihood ratio test, whereas the present analysis used 10 points. No other model selection criteria are described for the original analysis. The final original analysis model was 3 compartment, linear with no covariates. Between subject variability was included on clearance, Q3, and all volumes. Between occasion variability was described on central volume and Q2. The residual error model was proportional only. In comparison, the final model from this exercise was also 3 compartments, linear, with between subject variability on CL, Q2 and all volumes. One covariate was described, with central volume of distribution a power function of weight. The same 2 between occasion variability terms were identified, plus one additional, on clearance. The current final model had a combined proportional and additive residual error. The objective function value (OFV) of the original model was 8178.45, compared to the current analysis OFV of 8041.27 (delta OFV = 137.2 points), with 1 additional THETA, 1 additional OMEGA and 1 additional SIGMA parameters. This is consistent with the experience with ML methods to date, with the selection of a larger model than with traditional methods.

## Discussion

Each algorithm has a set of hyperparameters required. Some of these hyperparameters (e.g., population size) are common to all algorithms, but most are specific to the selected algorithm. Many of these hyperparameters can define the relative focus of the algorithm on “exploitation” vs “exploration”. Exploitation is the degree to which the selection of subsequent models to be run is based on what is already “known” and exploration is the degree to which the selection is outside of what is currently “known” to maximize the predictive uncertainty. In GA, the key parameter defining this exploitation/exploration tradeoff is the mutation rate. The mutation rate, as is the case in biology, introduces new content into the genome, which is unrelated to any previous genetic content and is random. If the mutation rate is low (even 0), little or no new genetic content will be introduced, and the only “evolution” will be from the crossover – basically re-shuffling the existing genetic contents (based on which features generate a good fitness value). Thus, no exploration would be done. If the mutation rate is high (e.g., 1.0), then no exploitation will be done, and the search is essentially a series of randomly selected models. The consequence of a tradeoff between exploitation and exploration is that, testing a hypothesis once (e.g., is this a two-compartment model vs a one-compartment model?) is helpful, but not confirmatory, and it therefore needs to be retested with different combinations of other features, as found by Wade et. al. [5] demonstrated. With each rejection of a hypothesis, the representation of that hypothesis (genetic content) in the population is reduced, but may be reintroduced at any time by mutation. Note that in traditional model selection, we commonly test any given hypothesis only once, then assume the test is correct regardless of other model features, corresponding to a very high exploitation component in model selection. In other words, we exploit what we believe to be “correct” without retesting. In the case of PSO, exploration is based on the random number introduced into the updated velocity (Eq. 1) with the exploitation vs. exploration based on the relative values of the coefficients for inertia/moving toward the best position in the population and the best position seen by that particle. Other algorithms have similar hyperparameters that can define this ratio of exploitation to exploration.

All ML algorithms implemented in pyDarwin package were able to find the optimal model structure searched by “gold standard” exhaustive search, which highlights the robustness of the machine learning application in nonlinear mixed effect model selection and optimization process. It’s noted that this analysis is based on a single sample model selection process, therefore inference on the robustness of the algorithm’s performance across various problems must be limited. To further evaluate the robustness of each ML algorithm, more datasets and corresponding exhaustive search results will be needed.

The most efficient algorithm measured by the number of unique models to the optimal is GP, which was able to find the optimal model after examining 495 models. However, it’s the least efficient algorithm based on the compute time, which is up to 2975.6 min. As what we have pointed out above, this analysis is based on a single, typical dataset, and changing the datasets, using different tokens, and adjusting the hyperparameters may impact the algorithm efficiency. We do, though based again on small sample size as well as other experience with this method, conclude that the two-bit local downhill search is likely critical to ensure robustness and should be done whenever the computation load is feasible.

The method implemented in pyDarwin is quite general, in that it simply manipulates text string. As such, it can readily be used for models other than PK. For example, searching different pharmacodynamic (PD) models (effect compartment, type 1–4 indirect response) can be accommodated. There are additional challenges to these models. One is execution time, as PD are frequently ordinary differential equations (ODEs) and they have a longer run times. This is a problem when inadequate computer resource is used, as typically hundreds to thousands of models are run, large scale parallel execution is typically needed. Fortunately, large scale parallel compute resources are readily available from cloud computer providers. Coding the different ODE models is more complex. “Other” type endpoints (e.g., categorical, survival) can be done as well, with similar challenges to ODE models.

## Conclusions

All algorithms, when combined with one- and two-bit local downhill search, were able to identify the optimal model in this single example. GA, RF, GP, and GBRT were able to identify the optimal model with only a one-bit local downhill search. In general, a two-bit local downhill search is recommended to ensure a robust search. GP was the most efficient based on the number of unique models to the optimal model measurement, while it took 2975.6 min to finally elapse. PSO was the least efficient, requiring 1710 model runs, when combined with two-bit local downhill search to find the optimal model.

## Supplementary Information

Below is the link to the electronic supplementary material.

Supplementary file1 (TXT 1 KB)

Supplementary file2 (TXT 5.45 KB)

Supplementary file3 (TXT 4.09 KB)

Supplementary file4 (TXT 3 KB)

Supplementary file5 (TXT 41.5 KB)

## Data Availability

No datasets were generated or analysed during the current study.
